# Identification of Paraptosis-Related Renal Cell Carcinoma Subtypes, Construction of a Prognostic Signature, and Determination of Tumor Microenvironment Landscape Using Bioinformatic Analysis and Experimental Verification

**DOI:** 10.3390/cimb48020233

**Published:** 2026-02-23

**Authors:** Mengyuan Qin, Meiting Chen, Yuling Gan, Xiangqian Feng, Ping Huang, Feifei Meng, Yufang Yang

**Affiliations:** School of Pharmacy, Guangxi Medical University, Nanning 530021, China; yfy108591@sr.gxmu.edu.cn (M.Q.); 202321052@sr.gxmu.edu.cn (M.C.); 202221102@sr.gxmu.edu.cn (Y.G.); 202321046@sr.gxmu.edu.cn (X.F.); 202321110@sr.gxmu.edu.cn (P.H.); 202421094@sr.gxmu.edu.cn (F.M.)

**Keywords:** paraptosis, renal cell carcinoma, tumor microenvironment, prognostic signature

## Abstract

Renal cell carcinoma (RCC) is a common and deadly urological cancer, for which there are no robust prognostic biomarkers or personalized treatment strategies. Paraptosis, a distinct form of regulated cell death marked by cytoplasmic vacuolization, is being increasingly recognized for its roles in tumorigenesis and therapy responses, yet its functional implications in RCC remain poorly defined. Transcriptomic profiles and corresponding clinical metadata from the TCGA-KIRC and GSE33371 datasets were systematically analyzed to characterize the paraptosis-related gene (PaRG) expression profile in renal cell carcinoma (RCC). Patients were categorized into two subtypes via consensus clustering, 574 overlapping differentially expressed genes (DEGs) were identified, and a four-gene (COL7A1, RNASE2, SLC10A2, and APOLD1) prognostic signature was constructed using LASSO and multivariate Cox regression. We analyzed the signature’s associations with tumor microenvironment (TME) features, cancer stem cell (CSC) indices, and tumor mutation burden (TMB), and validated the expression of the signature genes in RCC cell lines via qRT-PCR and Western blot. The four-gene signature showed robust prognostic performance (1-, 3-, and 5-year AUC: 0.751, 0.735, and 0.733 in the total cohort; 0.735, 0.731, and 0.767 in the training cohort), with high-risk patients having significantly poorer overall survival than the low-risk group. The low-risk group exhibited higher Stromal, Immune, and ESTIMATE scores (enriched immune/stromal infiltration), while the high-risk group had elevated CSC content and TMB, and the signature correlated with differential sensitivity to multiple chemotherapeutics. Both qRT-PCR and Western blot confirmed upregulation of COL7A1 and RNASE2 and downregulation of SLC10A2 and APOLD1 in RCC cell lines. Our study establishes a paraptosis-based two-subtype classification and four-gene prognostic signature for RCC that can reliably predicting patient survival, delineate TME characteristics, and guide personalized therapy, with COL7A1 emerging as a potential therapeutic target for advancing our understanding of paraptosis in RCC pathogenesis and optimizing treatment.

## 1. Introduction

Renal cell carcinoma (RCC) accounts for roughly 3% of adult malignancies and 80–90% of renal malignancies [1,2]. Global statistics recorded 431,288 new RCC cases and 179,368 RCC-related deaths in 2020; its disease burden continues to rise in developed regions, driven by lifestyle factors and improved diagnostic capacity [2,3,4]. While surgical resection/partial nephrectomy is curative for early RCC, most patients present at advanced stages, limiting treatments to tyrosine kinase inhibitors or immune checkpoint inhibitors [5,6,7]. Advanced RCC has dismal 5-year survival outcomes (12–15%), and patients exhibit significant prognostic heterogeneity. Conventional tools such as TNM staging fail to fully capture the intrinsic biological complexity of tumors, thereby limiting prognostic accuracy and the development of personalized therapeutic strategies, highlighting the urgent need to decode RCC’s genomic landscapes and develop robust models for prognosis prediction and therapeutic response assessment [8].

The progression and therapeutic outcomes of RCC are regulated by multiple biological factors [9]. (1) The tumor microenvironment (TME), particularly immune cell infiltration profiles and functional activity within tumor tissues, is a decisive factor in patient prognostic outcomes and responsiveness to immune checkpoint inhibitor therapy [10]. (2) Specific cancer stem cell (CSC) subsets drive tumorigenesis, metastasis, and drug resistance, with high CSC indices closely associated with aggressive phenotypes. (3) The tumor mutation burden (TMB) reflects tumor genomic instability and evolutionary potential. (4) the neoantigen load reflect patient responses to immunotherapeutic interventions [11]. Therefore, constructing a comprehensive analytical framework that integrates multiple aspects—including regulated cell death, the immune microenvironment, stem cell properties, and genomic variations—is crucial for achieving more precise prognostic stratification and treatment optimization.

Programmed cell death (PCD) is a core regulator of tumorigenesis and progression; the roles of pathways such as apoptosis, autophagy, and ferroptosis in RCC have been extensively studied [12,13,14]. Paraptosis—a caspase-independent, non-apoptotic PCD pathway that was first described in 2000—is characterized by endoplasmic reticulum/mitochondrial vacuolization and a lack of apoptotic body formation [15]. Emerging evidence links paraptosis to tumor growth inhibition (e.g., in lung cancer) and poor prognosis (e.g., in colorectal cancer), yet its role in RCC remains under-explored [16,17,18]. The molecular subtypes driven by paraptosis-related genes (PaRGs), their associations with the tumor microenvironment (TME), immune infiltration, and genomic mutations in RCC have not been characterized [17,18,19,20]. In addition, there is no prognostic model for RCC that considers paraptosis as the entry point and systematically links it to the RCC biological characteristics mentioned above to guide the clinical management of RCC.

In this study, we integrated transcriptomic and clinical data of RCC patients from the TCGA and GSE33371 datasets to dissect PaRG-mediated molecular patterns in RCC [21]. We first profiled PaRG expression and genetic variations, identifying two paraptosis-related molecular subtypes (PaRG cluster A/B) whose pathway enrichment (via GSVA) and immune infiltration (via ssGSEA) profiles were then characterized. Next, we screened overlapping differentially expressed genes (DEGs) to construct a four-gene prognostic risk model, validated its predictive performance across cohorts, and linked it to TME features, cancer stem cell (CSC) indices, and tumor mutation burden (TMB). Finally, we verified the expression of the signature genes (COL7A1 RNASE2, SLC10A2, and APOLD1) in clinical samples and cell lines using qPCR and explored COL7A1’s functional role in RCC cells. Our findings demonstrate the role of paraptosis in RCC and provide a theoretical basis for individualized diagnosis and treatment.

## 2. Materials and Methods

### 2.1. RCC Dataset and Preprocessing

The RNA-sequencing and corresponding clinical data of 530 RCC cases and 70 healthy cases were downloaded from the TCGA database (https://portal.gdc.cancer.gov/, accessed on 21 October 2025). The RCC gene expression profiles and clinical characteristics from the GSE33371 dataset (n = 65) were obtained from the GEO database (https://www.ncbi.nlm.nih.gov/geo/, accessed on 21 October 2025) [21]. Gene symbols were converted from the probes based on the corresponding platform annotation files (e.g., GPL570 for GSE33371). Patients with missing survival information were excluded. The TCGA and GSE33371 datasets were merged using the “sva” R package to remove batch effects [22]. Information on 32 paraptosis-related genes (PaRGs) was obtained from GeneCards (https://www.genecards.org/, accessed on 21 October 2025). Copy number variation (CNV) data was downloaded from UCSC Xena (https://xenabrowser.net/, accessed on 21 October 2025).

### 2.2. Differential Expression Gene and Consensus Clustering Analysis of PaRGs

Wilcoxon rank-sum test (via “limma” package) was used to analyze differential PaRG expression between RCC and healthy samples (DEGs selected based on *p*-value < 0.05) [23]. Consensus clustering (via “ConsensusClusterPlus” R package) was applied to categorize RCC patients into 2 distinct molecular subtypes (PaRG cluster A/B) based on PaRGs expression; 1000 repetitions were performed to ensure robustness [24]. The optimal subtype number (k = 2) was determined by evaluating the Cumulative Distribution Function (CDF) and CDF Delta area. Principal Component Analysis (PCA; via “ggplot2”) confirmed the differential transcriptome profiles between the 2 subgroups [25].

### 2.3. Gene Set Variation Analysis and Functional Enrichment Analysis

The “GSVA” R package was used to perform GSVA (based on KEGG gene set c2.cp.kegg.symbols.gmt from MSigDB) to detect biological functions that can distinguish between the 2 paraptosis subtypes (in TCGA and GSE33371 cohorts) [26,27]. “clusterProfiler” was used for KEGG/GO analysis; pathways with *p* < 0.05 and logFC > 0.5 were considered statistically significant [28].

### 2.4. Construction of Paraptosis Risk Model

A total of 530 RCC patients were randomly split into testing/training groups (1:1 ratio). A total of 574 overlapping DEGs were identified via pairwise comparisons of the 2 paraptosis subtypes (threshold: Log_2_ (fold change) > 0.585, adjusted *p*-value < 0.05). Univariate Cox regression identified 87 DEGs associated with RCC prognosis. LASSO Cox regression was used to mitigate overfitting, and multivariate Cox regression was used to select the optimal genes for the risk model: Paraptosis score = Σ_ni=1_exp(X_i_) × coef(X_i_) (exp(X_i_) = gene expression level; coef(X_i_) = regression coefficient) [29]. Patients were stratified into high/low-risk groups based on the median risk score. Time-dependent ROC analysis was used to assess signature performance; 1000 bootstrap resamplings were performed to validate the test set.

### 2.5. Assessment of Tumor Microenvironment

The “ESTIMATE” package was used to calculate the Immune, Stromal, and ESTIMATE scores for each sample. Wilcoxon tests were used to analyze score differences between risk groups [30]. Single-sample GSEA (ssGSEA; via “gsva”) was used to quantify immune cell subset enrichment in individual samples; correlations between risk genes and immune cells were analyzed via “limma” and “ggplot2” [26].

### 2.6. Survival Analysis of RCC

Kaplan–Meier survival plots (generated using the “survminer” and “survival” packages) were constructed to visualize the OS differences between subtypes (PaRG cluster, geneCluster, risk score); log-rank tests were used to assess significance [31].

### 2.7. Development of Nomograms

Nomograms (generated using the “rms” package) integrated clinical characteristics (gender, age, and stage) and risk score to predict 1-, 3-, and 5-year OS [32]. Each variable was assigned a score; cumulative scores reflected prognostic risk. Calibration plots were generated to evaluate the concordance between predicted and actual OS.

### 2.8. Tumor Mutation Burden and Cancer Stem Cell Index

The tumor mutation burden (TMB) and mutation landscapes of the high-risk (n = 169) and low-risk (n = 189) groups were analyzed via “maftools” [33]; genes with a mutation frequency >5% were defined as having a high frequency of mutations. Spearman correlation was used to analyze the association between risk score and cancer stem cell index.

### 2.9. Cell Culture, RNA Extraction, and Quantitative Real-Time PCR

HK-2 cells were provided by Procell Life Science & Technology Company (Batch No. CL-0109; Wuhan, China). 786-O and ACHN cells were provided by Wei Li (Nanning, China). HK-2 cells were cultured in MEM (Gibco, Cat No. 41500034, New York, NY, USA) at 37 °C (5% CO_2_). 786-O and ACHN cells were cultured in RPMI-1640 (Solarbio, Cat No. 31800, Beijing, China) at 37 °C (5% CO_2_). Total RNA was isolated using FreeZol Reagent (Vazyme, Cat No. R711-01, Nanjing, China); reverse transcription (Vazyme, Cat No. Q222) and qRT-PCR (Vazyme, Cat No. MQ102-01) were performed to quantify the expression of COL7A1, RNASE2, SLC10A2, and APOLD1 (with β-actin as the internal control). The PCR primers used were manufactured by Sangon Biotech (Shanghai, China). The primer sequences are provided in the Supplementary Materials. The 2^−ΔΔCT^ method was used to calculate the relative expression of the target genes [34].

### 2.10. Western Blot

The four-gene signature was validated by assessing COL7A1, RNASE2, SLC10A2, and APOLD1 expression levels in HK-2, 786-O, and ACHN cell lines via Western blot. The HK-2, 786-O and ACHN cells were homogenized in chilled RIPA extraction solution (Beyotime, Cat No. P0013B, Shanghai, China) supplemented with a freshly prepared cocktail of protease inhibitors (Solarbio, Cat No. 329-98-6, Beijing, China). The protein concentration was quantified using a caprylic acid assay kit (Beyotime, Cat No. P0010). Equal quantities of protein were subjected to separation via SDS-PAGE and subsequently blotted onto a PVDF membrane. The membrane was then incubated with primary antibodies targeting COL7A1 (Proteintech, Cat No. 19799-1-AP, Wuhan, China), RNASE2 (Proteintech, Cat No. 18172-1-AP), SLC10A2 (Proteintech, Cat No. 25245-1-AP), APOLD1 (Biorbyt, Cat No. orb325529, Cambridge, UK), and β-actin (Proteintech, Cat No. 66009-1-Ig) at 4 °C overnight. Next, the membrane was incubated with a horseradish peroxidase-conjugated secondary antibody at ambient temperature for 1 h.

### 2.11. Statistical Analysis

Statistical analyses were performed using R software (v4.4.2 and v4.3.0; packages included ConsensusClusterPlus, survival, limma, GSVA, and maftools) and GraphPad Prism 9. Continuous data are presented as the mean ± standard deviation (if normally distributed) or median and interquartile range (if non-normally distributed). Group comparisons were conducted with Student’s *t*-test (normally distributed two-group data), the Wilcoxon rank-sum test (non-normally distributed two-group data), or the Kruskal–Wallis test (multi-group data). Survival differences between subgroups (e.g., PaRG cluster and risk groups) were evaluated via Kaplan–Meier curves and log-rank tests. Spearman’s rank correlation was used to assess associations between risk scores and immune cell abundance or cancer stem cell (CSC) indices. Consensus clustering (ConsensusClusterPlus package) was repeated 1000 times for robustness; LASSO Cox regression employed 10-fold cross-validation to select the optimal penalty parameter (λ) and avoid overfitting. Statistical significance was defined as *p* < 0.05; all in vitro experiments were independently performed three times.

## 3. Results

### 3.1. Differential Expression of and Genetic Variation in PaRGs in RCC

**Figure 1:**
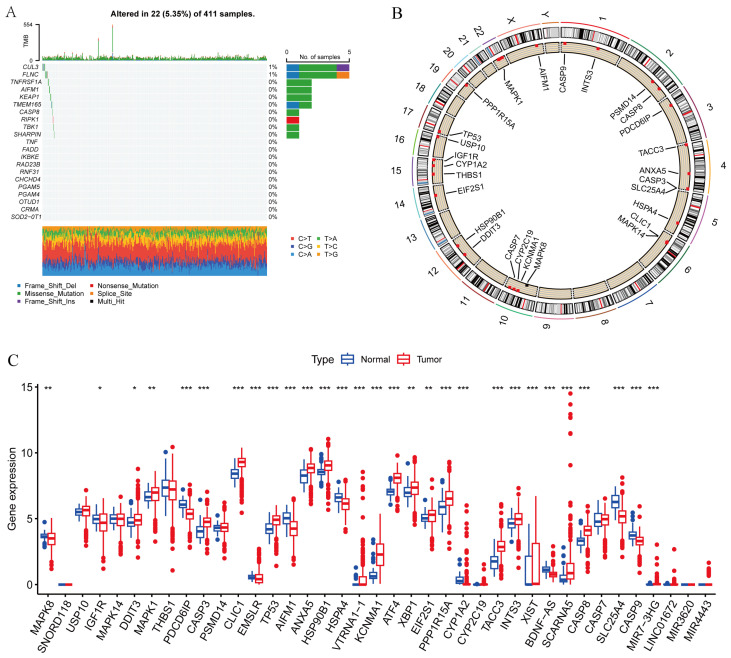
Expression of and genetic alterations in paraptosis-related genes (PaRGs) in renal cell carcinoma (RCC). (**A**) The maftools plot shows the incidence of somatic mutations in PaRGs (22 of 411 RCC samples (5.35%)) with different mutation types and nucleotide changes indicated by legends. (**B**) The circular plot shows the chromosomal location of the CNV alterations in the PaRGs. (**C**) The box plot presents the expression levels of the PaRGs in RCC (Tumor, red) vs. normal tissues (Normal, blue). PaRGs: paraptosis-related genes; RCC: renal cell carcinoma; CNV: copy number variation. * *p* < 0.05, ** *p* < 0.01, *** *p* < 0.001.

We first collected paraptosis-related genes (PaRGs) for analysis. As shown in Figure 1A, 22 (5.35%) of 411 RCC samples had somatic mutations in PaRGs. The copy number variation (CNV) analysis results in Figure 1A show the number of samples with CNV alterations in individual PaRGs. The chromosomal locations of the CNV alterations in the PaRGs are displayed in Figure 1B. MAPK1 and CLIC1 were found to be upregulated in RCC while PDCD6IP, EMSLR, and BDNF-AS exhibited marked downregulation (Figure 1C).

### 3.2. Identification of PaRG Clusters in RCC

**Figure 2:**
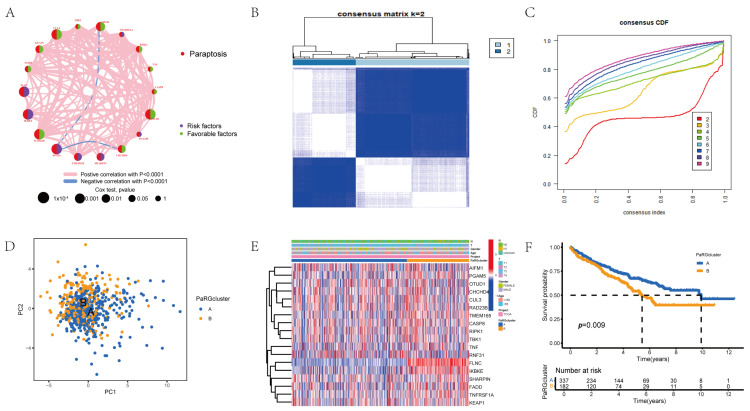
Identification of molecular subtypes of PaRGs in RCC. (**A**) A network plot showing the correlations between PaRGs (labeled as “Oxeiptosis”) and risk/favorable factors: pink lines indicate positive correlations (*p* < 0.0001), light blue lines indicate negative correlations (*p* < 0.0001), and black dots represent Cox test *p*-values. (**B**) Consensus matrix heatmap showing 2 clusters (k = 2) (blue/white blocks represent sample clustering consistency). (**C**) Consensus CDF plot showing the cumulative distribution function of consensus indices with different cluster numbers (k = 2~9). (**D**) PCA diagram of RCC samples in 2 clusters (PaRG cluster A: blue; B: orange). (**E**) Complex heatmap showing correlations between PaRG expression (red/blue for high/low expression) and clinical features (e.g., “Gender”) in the 2 clusters. (**F**) Survival analysis of the 2 PaRG clusters (A: blue; B: orange) with (*p* = 0.009) “Number at risk” given in table below.

To explore the interaction, correlation, and prognostic relevance of PaRGs, a network map was constructed (Figure 2A), which displays the correlations between PaRGs (labeled “Oxeiptosis”) and risk/favorable factors, with pink/light blue lines representing positive/negative correlations (both *p* < 0.0001), and black dots indicating Cox test *p*-values. We performed unsupervised clustering of the RCC patients based on PaRG expression; the consensus matrix (Figure 2B) and consensus CDF plot (Figure 2C) indicated that the optimal clustering result was obtained when k = 2 (among k = 2~9). Thus, RCC patients were categorized into two clusters (PaRG cluster A: blue; B: orange). Principal component analysis (PCA; Figure 2D) showed clear separation between the two clusters. The complex heatmap in Figure 2E displays the PaRG expression patterns (red = high, blue = low) and their correlation with clinical features in the two clusters. Kaplan–Meier survival analysis (Figure 2F) revealed significant differences in overall survival (OS) between the two clusters (*p* = 0.009), with distinct survival probabilities for PaRG clusters A and B.

**Figure 3:**
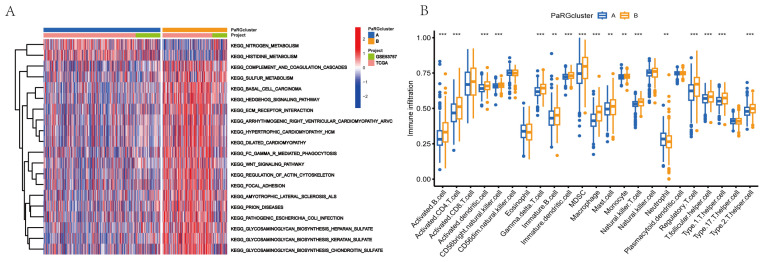
GSVA pathway and immune infiltration profiles of PaRG clusters. (**A**) GSVA heatmap showing pathway enrichment differences (red = high; blue = low) between 2 PaRG clusters (A/B) in GSE33371/TCGA cohorts. (**B**) ssGSEA boxplot showing immune cell infiltration differences between PaRG cluster A (blue) and B (orange); ** *p* < 0.01, *** *p* < 0.001.

To further characterize the two PaRG clusters (A/B), we performed GSVA and ssGSEA. GSVA revealed distinct pathway enrichment patterns between PaRG clusters A and B (Figure 3A), with several pathways (e.g., KEGG_NITROGEN_METABOLISM) showing differential activation between the two clusters (in both GSE58575 and TCGA cohorts). ssGSEA of immune cell infiltration (Figure 3B) demonstrated significant differences between PaRG cluster A (blue) and B (orange): most immune cell subsets (e.g., activated CD4 T cells and macrophages) exhibited higher infiltration in PaRG cluster B, while eosinophils showed relatively higher enrichment in PaRG cluster A.

### 3.3. Identification of PaRG Signatures in RCC

**Figure 4:**
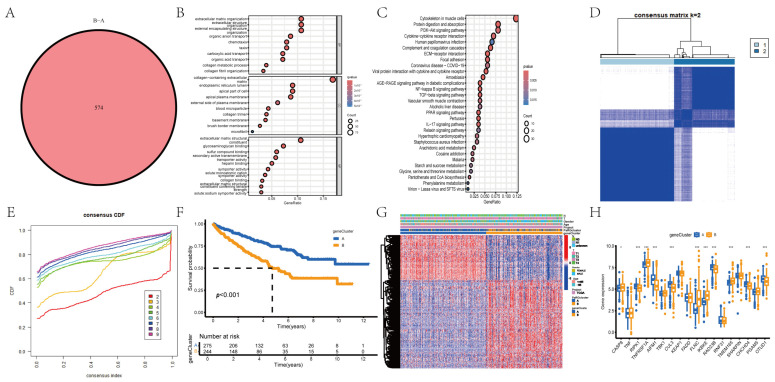
Functional enrichment and gene cluster analyses of PaRGs. (**A**) Venn diagram showing 574 overlapping DEGs between 2 PaRG clusters. (**B**) GO enrichment plot showing enriched BP (e.g., extracellular matrix organization), CC (e.g., membrane structures), and MF (e.g., transporter activity) terms among DEGs. (**C**) KEGG enrichment plot showing DEG-associated pathways (e.g., PI3K–Akt signaling). (**D**) Consensus CDF plot showing the optimal clustering (k = 2) for gene clusters. (**E**) Consensus matrix heatmap showing the clustering consistency of 2 gene clusters (A/B). (**F**) Survival curves showing OS differences between gene clusters (*p* = 0.001); “Number at risk” table is included under the graph. (**G**) Complex heatmap showing DEG expression patterns + clinical/cohort features. (**H**) Boxplot showing gene expression differences between gene clusters A (blue) and B (orange). * *p* < 0.05, *** *p* < 0.001.

We performed pairwise differential expression analysis of the two PaRG clusters and identified 574 overlapping DEGs, as shown in the Venn diagram (Figure 4A). GO enrichment analysis (Figure 4B) identified enrichment of these DEGs within key biological processes (e.g., extracellular matrix organization), cellular components (e.g., cell membrane structures), and molecular functions (e.g., transporter activity). KEGG analysis (Figure 4C) revealed enrichment of pathways such as PI3K–Akt signaling and ECM–receptor interactions. Univariate Cox regression of DEG expression was used to stratify the patients into two gene clusters (A/B; Figure 4D,E). Kaplan–Meier analysis (Figure 4F) revealed that gene cluster B was associated with markedly superior overall survival compared to A (*p* = 0.001). The complex heatmap in Figure 4G integrates clinical features (e.g., cohort) and gene expression profiles. Most genes were upregulated in gene cluster A and downregulated in B. Transcriptomic profiling (Figure 4H) further confirmed the differential expression of the key genes between the two gene clusters.

### 3.4. Construction of Prognostic Risk Scoring Model Based on PaRG Expression Profile

**Figure 5:**
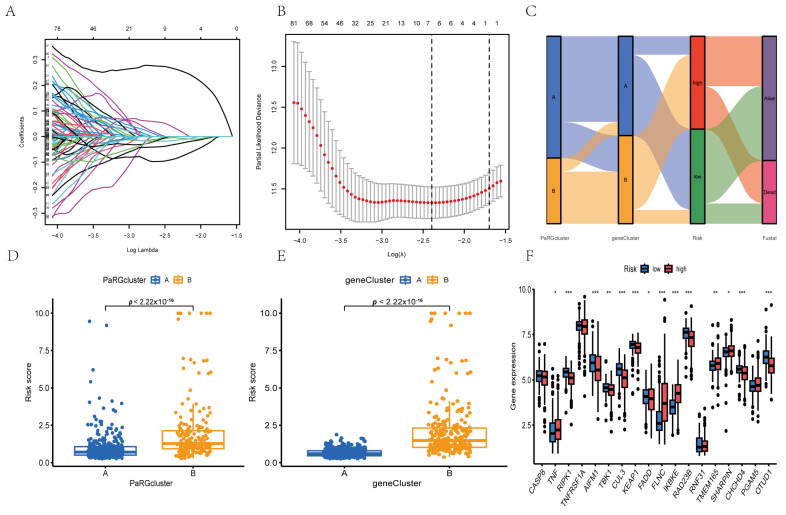
PaRG-based risk score model construction and correlation analyses. (**A**) LASSO regression coefficient profile of prognostic genes. (**B**) Partial likelihood deviance plot for LASSO model optimization. (**C**) Sankey plot showing correlations between PaRG cluster, gene cluster, risk group, and survival status. (**D**) Boxplot showing risk score differences between 2 PaRG clusters (*p* < 2.22 × 10^−16^). (**E**) Boxplot showing risk score differences between 2 gene clusters (*p* < 2.22 × 10^−16^). (**F**) Differential gene expression profiles between high- and low-risk groups. * *p* < 0.05, ** *p* < 0.01, *** *p* < 0.001.

The RCC patients were split into training and test sets (265 samples each). Univariate Cox regression of 142 DEPaRGs identified 87 prognosis-related genes (*p* < 0.05) in the training set. LASSO regression (1000 iterations, 10-fold cross-validation) refined these to identify the key genes (Figure 5A,B). A risk score model was constructed (via multivariate Cox regression), with the patients stratified into high- and low-risk subgroups based on median risk score. The Sankey diagram in Figure 5C shows the risk score distribution across different PaRG clusters, gene clusters, and survival statuses. The boxplots in Figure 5D,E reveal significantly higher risk scores in PaRG cluster B and gene cluster B (*p* < 2.22 × 10^−16^). Differential expression analysis (Figure 5F) demonstrated upregulation of multiple genes (e.g., FLNC and TMEM165) in the high-risk group.

### 3.5. Validation of Prognostic Risk Scoring Model Based on PaRG Expression Profile

**Figure 6:**
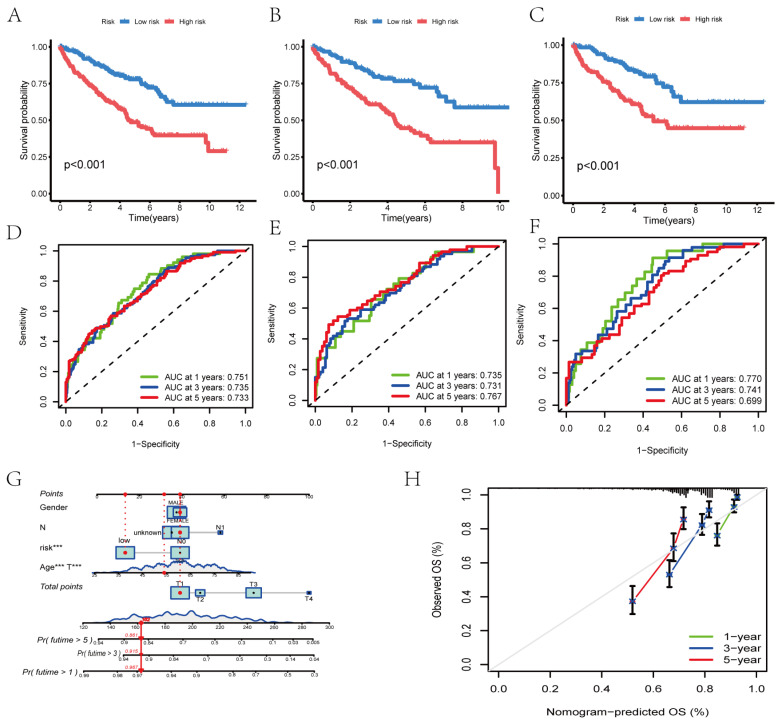
Validation of the PaRG-based prognostic signature. (**A**–**C**) Kaplan–Meier survival curves showing that low-risk patients had better OS than high-risk patients in the total, training, and testing sets (*p* < 0.001). (**D**–**F**) ROC curves showing 1-, 3-, and 5-year OS prediction AUCs (e.g., total set: 0.751, 0.735, and 0.733). (**G**) Nomogram of integrated risk score + clinical features (age, gender, and stage) for RCC survival prediction. (**H**) Calibration curves showing consistency between nomogram-predicted and observed 1-, 3-, and 5-year OS. *** *p* < 0.001.

Kaplan–Meier analysis revealed that the low-risk patients had significantly better survival than the high-risk patients in the total, training, and testing sets (*p* < 0.001; Figure 6A–C). ROC curve analysis was performed to demonstrate the predictive accuracy of the risk score: in the total set, 1-, 3-, and 5-year AUCs were 0.751, 0.735, and 0.733; in the training set, they were 0.735, 0.731, and 0.767; and in the testing set, they were 0.770, 0.741, and 0.699 (Figure 6D–F), indicating favorable prognostic performance. We then constructed a nomogram integrating risk score, gender, age, and clinical stage (Figure 6G). The calibration curve showed good consistency between nomogram-predicted and observed overall survival (OS) (Figure 6H), confirming the nomogram’s validity for RCC survival prediction.

**Figure 7:**
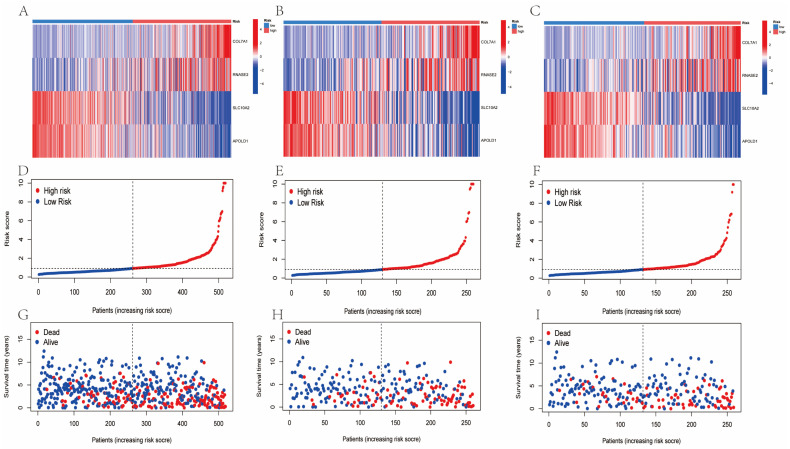
Risk gene expression, score distribution, and survival status in RCC. (**A**–**C**) Heatmaps showing expression of 4 risk genes (COL7A1, RNASE2, SLC10A2, and APOLD1) in high- and low-risk groups (total, training, and testing sets). (**D**–**F**) Risk score distribution showing sharp increase at median cutoff (dashed line) separating low- (blue) and high-risk (red) patients (total, training, and testing sets). (**G**–**I**) Survival time scatter plots showing that high-risk patients (red) have shorter survival times compared to low-risk patients (blue) (total, training, and testing sets).

In the total, training, and testing sets (Figure 7A–C), COL7A1 and RNASE2 were found to be upregulated in the high-risk group, while SLC10A2 and APOLD1 were downregulated. The risk score plots revealed a sharp increase in risk score at the median cutoff (separating low- and high-risk patients) in all sets. Survival time scatter plots further demonstrated an inverse correlation between risk score and survival time, alongside a positive association between risk scores and mortality rate: high-risk patients (red) had shorter survival times and higher mortality compared to low-risk patients (blue). These results confirm that elevated risk scores correlate with poorer RCC survival outcomes (Figure 7D–I).

### 3.6. Relationship Between Signature Genes and TME

**Figure 8:**
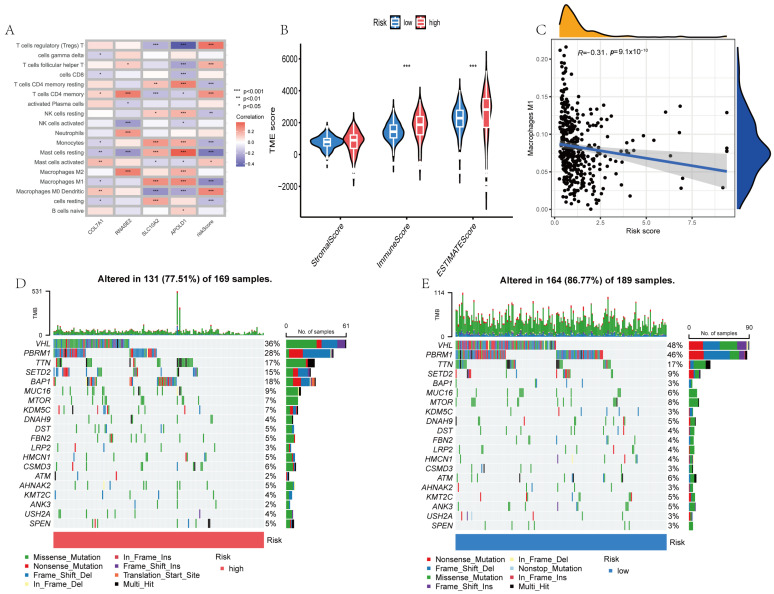
Prognostic signature associations with TME, CSC index, and TMB. (**A**) Correlation heatmap of 4 signature genes vs. immune cell abundance (e.g., COL7A1 with neutrophils). (**B**) Boxplots showing that the low-risk group has higher Stromal, Immune, and ESTIMATE scores compared to the high-risk group (*** *p* < 0.001). (**C**) Scatter plot showing that risk score negatively correlates with M1 macrophages (r = −0.31, *p* = 1 × 10^−10^). (**D**,**E**) Mutation landscapes showing that 77.51% and 86.77% of the genes were altered in the high- and low-risk groups. Top genes included VHL and PBRM1.

Correlation analysis (Figure 8A) found that the four risk genes (COL7A1 RNASE2, SLC10A2, and APOLD1) were associated with multiple immune cells (e.g., neutrophils and M2 macrophages). The low-risk group had significantly higher Stromal, Immune, and ESTIMATE scores compared to the high-risk group (Figure 8B). Risk score positively correlated with cancer stem cell content (e.g., M1 macrophages: r = −0.31, *p* = 1 × 10^−10^; Figure 8C). Somatic mutation analysis (Figure 8D,E) revealed that the top mutated genes (VHL, PBRM1, SETD2, etc.) were shared between the high- (77.51% altered) and low-risk (86.77% altered) groups, though the mutation frequencies differed (e.g., VHL: 36% in high-risk vs. 48% in low-risk group).

### 3.7. Experimental Validation of Signature Genes and Biological Function of COL7A1

**Figure 9:**
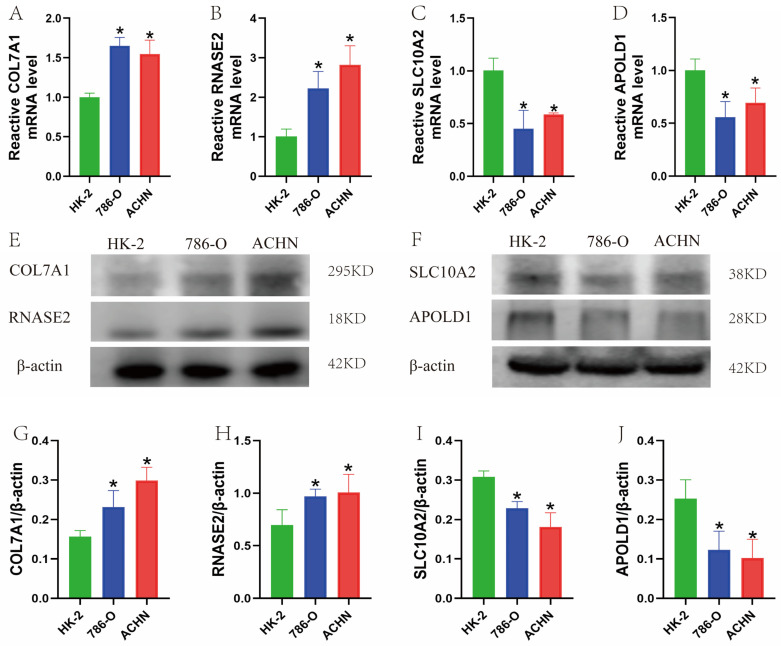
Validation of signature gene expression in cell lines. (**A**–**D**) qRT-PCR and (**E**–**J**) Western blot analyses of the four signature genes in RCC cell lines (786-O and ACHN) and normal HK-2 cells. * *p* < 0.05 vs. HK-2.

qRT-PCR analysis of renal cell carcinoma (RCC) cell lines (786-O and ACHN) and the normal renal epithelial cell line HK-2 confirmed that COL7A1 and RNASE2 were significantly upregulated in RCC cell lines (*p* < 0.001) (Figure 9A,B), while SLC10A2 and APOLD1 were significantly downregulated (*p* < 0.001) (Figure 9C,D). Western blot further verified that COL7A1 and RNASE2 were upregulated (Figure 9G,H), whereas SLC10A2 and APOLD1 were downregulated in RCC cell lines (Figure 9I,J).

## 4. Discussion

Renal cell carcinoma (RCC) is an aggressive urological malignancy with a growing global disease burden, inflicting substantial harm to worldwide public health and economic systems [5,35,36]. Even with the advancements in diagnostic and treatment approaches, most patients are diagnosed at an advanced stage (where surgical resection is not feasible), leading to poor prognosis. Thus, there is an urgent need to explore RCC progression mechanisms and identify reliable prognostic biomarkers/therapeutic targets [37,38].

Paraptosis, an emerging programmed cell death pathway that is independent of apoptosis, contributes to tumorigenesis and anti-tumor therapy [17,39], although its functional relevance in RCC has yet to be defined. Here, we systematically analyzed 32 paraptosis-related genes (PaRGs) in RCC (expression/genetic variations), identified two paraptosis-related molecular subtypes, constructed a prognostic signature, and explored its associations with the tumor microenvironment (TME), cancer stem cell (CSC) content, and tumor mutation burden (TMB). Our findings clarify the role of paraptosis in RCC and lay a foundation for personalized treatment.

We first analyzed somatic mutations, copy number variations (CNVs), and differential expression of 32 PaRGs in RCC: most PaRGs were found to be upregulated in RCC tissues, while SLC10A2 and APOLD1 were downregulated. Our findings suggest PaRGs may drive RCC progression via genetic mutations and abnormal expression.

Consensus clustering stratified the RCC patients into two paraptosis-related subtypes (PaRG clusters A and B), and unsupervised clustering of 574 overlapping DEGs (between subtypes) yielded two gene clusters (A and B). Survival analysis showed that gene cluster B was associated with better OS compared to gene cluster A. GSVA and ssGSEA revealed that the low-risk group (linked to favorable prognosis) had higher Stromal, Immune, and ESTIMATE scores, indicating richer stromal/immune cell infiltration, which is consistent with stronger anti-tumor immune potential.

We constructed a prognostic signature using four genes (COL7A1, RNASE2, SLC10A2, APOLD1), which showed robust prognostic value in the training and testing sets: high-risk patients were consistently found to have poorer OS. The nomogram (integrating signature + clinical features) exhibited good predictive accuracy, offering a practical clinical prognosis tool.

These four signature genes are linked to tumor progression. COL7A1 (an ECM component) promotes cell adhesion/migration/invasion [40]. It is generally weakly expressed in tumor tissue (in approximately 70% of patients), with high expression serving as a robust indicator of poor prognosis—which has been consistently reported in independent cohorts of different ethnic groups. Its expression is inversely correlated with overall survival, supporting its utility as either a binary (low/high expression) or continuous prognostic factor [41].

RNASE2 (ribonuclease A superfamily) may regulate the immune microenvironment to drive RCC growth. It is closely associated with immunomodulation, participating in antigen processing and presentation, B cell receptor signaling, natural killer cell-mediated cytotoxicity, and T cell receptor signaling pathways [42]. As a key mediator of human immune function (alongside RNASE3), RNASE2 plays a critical role in pathogen sensing via toll-like receptor 8 (TLR8) [43]. Notably, combined analysis of RNASE2 with six other immune-related genes has demonstrated prognostic value in ccRCC [44], while its correlation with EZH2—which is involved in cell cycle regulation, DNA damage repair, and JAK–STAT and WNT signaling—further underscores its involvement in tumor-promoting pathways [42].

SLC10A2 (bile acid transporter) downregulation correlates with poor prognosis. This gene encodes the apical sodium-dependent bile acid transporter (ASBT) that localizes to the distal ileum and renal proximal tubule luminal membrane, where it is essential for bile acid metabolism and enterohepatic bile acid–cholesterol homeostasis [45]. Its downregulation increases fecal bile acid secretion, promoting tumorigenesis. It is also a primary epigenetic target of KDM6B, which regulates its expression via H3K27me3 demethylation of its promoter [46]. SLC10A2 activates intracellular signaling pathways (PKC, PI3K, MAP kinase, and ERK), with ERK propagating signals through the transcription factor AP1 [47,48]. Critically, it mediates KDM6B’s immunomodulatory functions: restoring SLC10A2 expression normalizes ERK/AP1 activation and CXCL secretion, reversing the MDSC recruitment and immunosuppression induced by KDM6B deficiency. Additionally, SLC10A2 correlates positively with CD4/CD8 T cell infiltration, indicating that it is involved in immune microenvironment regulation and can be used for prognosis prediction in colorectal cancer (COAD). Genetic perturbation studies in MC38 cells have confirmed its core role in regulating immunosuppressive chemokine production [46].

APOLD1 (Apolipoprotein L Domain-Containing 1, a secreted protein) inhibits angiogenesis; its downregulation may facilitate RCC growth/metastasis. As an endothelial cell early response protein, it plays pivotal roles in regulating endothelial signaling pathways and vascular function [49]. APOLD1 organizes endothelial cell–cell junctions via cytoskeletal interactions [50], a process that promotes angiogenesis—though it is dispensable for normal development [51]. This intercellular junction activity is hypothesized to involve membrane fusion, linking its structural function to vascular remodeling in tumor progression.

Tumor-associated macrophages (TAMs) represent the most abundant immune cell population within the RCC TME, accounting for over 50% of the total tumor volume. Derived from circulating monocytes and tissue-resident M0 macrophages and recruited and polarized by tumor-derived chemokines, cytokines, and metabolites, TAMs can adopt two functionally divergent phenotypes with opposing roles in tumor biology. Classically activated M1 TAMs, or “killer macrophages,” exhibit robust phagocytic activity and exert pro-inflammatory, anti-tumor effects by inhibiting cancer cell proliferation and causing tissue damage [52]. In contrast, alternatively activated M2 TAMs, referred to as “repair macrophages,” mediate anti-inflammatory responses, promote cancer cell proliferation and tissue repair, and drive pro-tumorigenic processes [53]. In the context of RCC, these two TAM subsets play diametrically opposed roles in tumor progression: M1 TAM infiltration correlates negatively with tumor metastasis and TNM stage, and higher M1 abundance is associated with improved overall survival in RCC patients. Conversely, M2 TAMs are the predominant TAM subtype in RCC and are strongly linked to poor clinical prognosis. Mechanistically, M2 TAMs secrete C-X-C motif chemokine ligand 13 (CXCL13), which potentiates RCC cell invasion, migration, and epithelial–mesenchymal transition (EMT) [54], and increased M2 TAM infiltration has also been associated with RCC recurrence. Collectively, these observations highlight M2 TAMs as a promising potential target for RCC immunotherapy [55].

TME analysis showed that the risk score was negatively correlated with M1 macrophages (anti-tumor immune cells; r = −0.31, *p* = 1 × 10^−10^) and positively correlated with pro-tumor immune subsets (e.g., M2 macrophages). The somatic mutation landscapes revealed that 77.51% and 86.77% of the genes were altered in the high- and low-risk groups. The top mutated genes (VHL, PBRM1, and SETD2) were shared between the two groups. The risk score was also positively correlated with CSC content, linking higher risk scores to tumor recurrence and drug resistance. These findings suggest this signature can be used to accurately predict the TME composition and CSC content, and is associated with TMB, and thus can be used to guide immunotherapy and targeted therapy.

Despite obtaining meaningful findings, this study has limitations: it used data from public databases, which require prospective clinical validation; a small in vitro sample size was used and should be expanded in future studies; and the roles of paraptosis and the signature genes in RCC require deeper in vivo and in vitro exploration.

## 5. Conclusions

We identified two paraptosis-related RCC subtypes and constructed a prognostic signature with strong prognostic value (by predicting TME composition, CSC content, and TMB). COL7A1 emerged as a potential therapeutic target as it promotes RCC cell proliferation, migration, and invasion. Our findings enhance our understanding of paraptosis in RCC and provide new strategies for personalized diagnosis and treatment.

## Data Availability

The data presented in this study are openly available from TCGA (https://portal.gdc.cancer.gov, accessed on 2 October 2025), GEO (https://www.ncbi.nlm.nih.gov/geo/, accessed on 21 October 2025), GeneCards (https://www.genecards.org, accessed on 21 October 2025), and UCSCXena (https://xenabrowser.net, accessed on 21 October 2025).

## Supplementary Materials

The following supporting information can be downloaded at: https://www.mdpi.com/article/10.3390/cimb48020233/s1.
