# Supplementary material for: Identification of Paraptosis-Related Renal Cell Carcinoma Subtypes, Construction of a Prognostic Signature, and Determination of Tumor Microenvironment Landscape Using Bioinformatic Analysis and Experimental Verification
Source: Curr Issues Mol Biol. 2026 Feb 23;48(2):233. doi: 10.3390/cimb48020233 (PMC12939344; doi:10.3390/cimb48020233)
Supplement: Supplementary file 1 [file cimb-48-00233-s001.zip › Original Western Blots.pdf]

Fig9

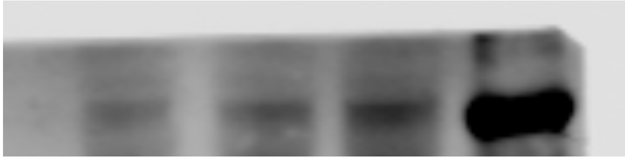

COL7A1

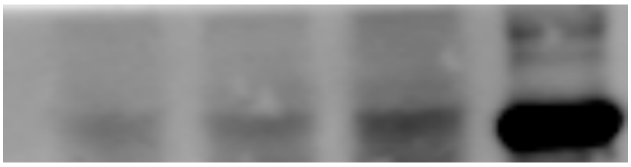

COL7A1

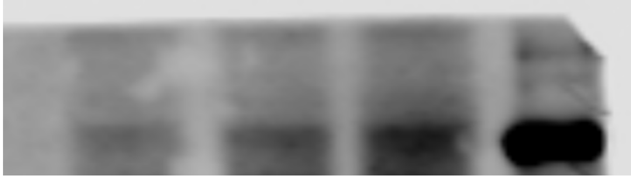

COL7A1

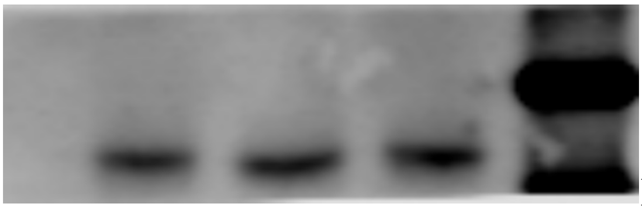

RNASE2

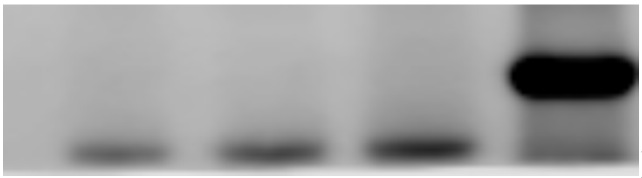

RNASE2

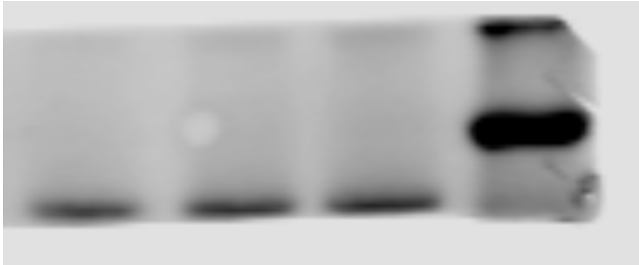

RNASE2

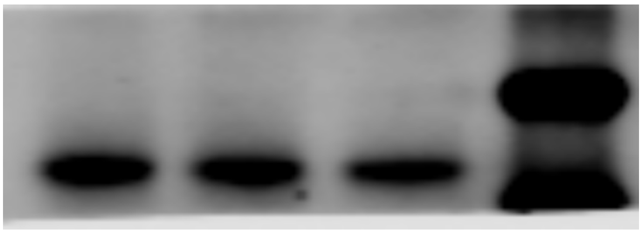

COL7A1/RNASE2_β-actin

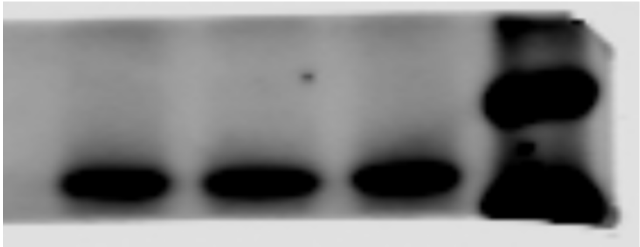

COL7A1/RNASE2_β-actin

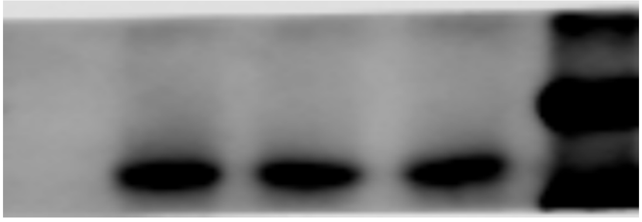

COL7A1/RNASE2_β-actin

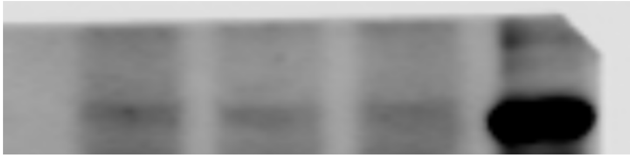

SLC10A2

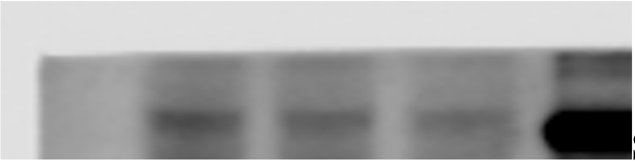

SLC10A2

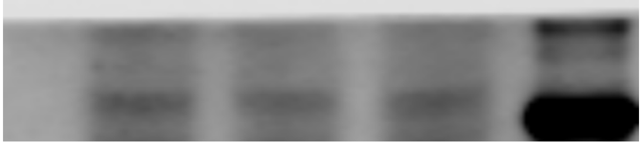

SLC10A2

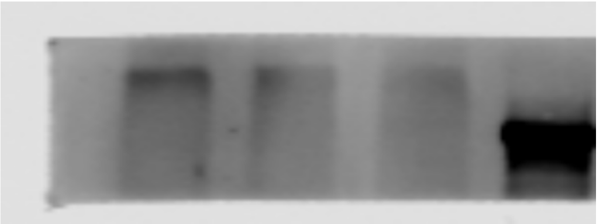

APOLD1

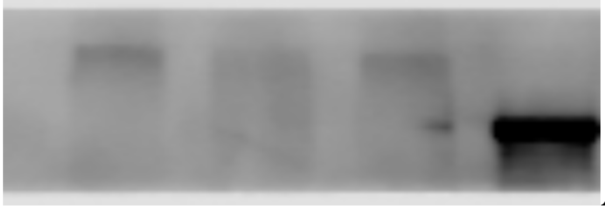

APOLD1

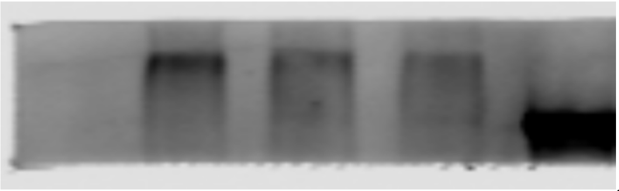

APOLD1

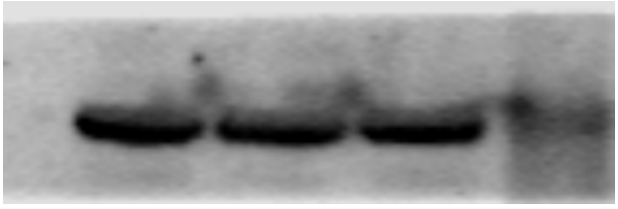

SLC10A2/APOLD1_β-actin

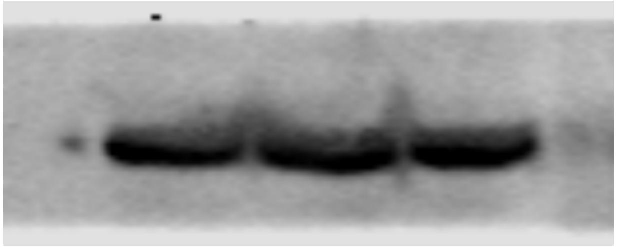

SLC10A2/APOLD1_β-actin

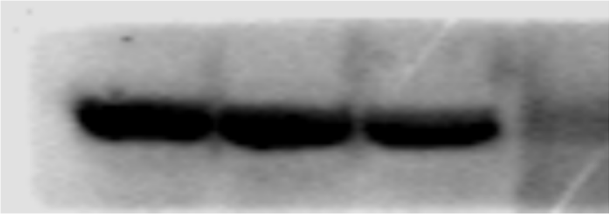

SLC10A2/APOLD1_β-actin
