# Supplementary material for: Identification of Paraptosis-Related Renal Cell Carcinoma Subtypes, Construction of a Prognostic Signature, and Determination of Tumor Microenvironment Landscape Using Bioinformatic Analysis and Experimental Verification
Source: Curr Issues Mol Biol. 2026 Feb 23;48(2):233. doi: 10.3390/cimb48020233 (PMC12939344; doi:10.3390/cimb48020233)
Supplement: Supplementary file 1 [file cimb-48-00233-s001.zip › supplementary.pdf]

Supplementary Table S1. The sequences of primers.

Oligonucleotide	Sequences
Human COL7A1 primer	F: 5'-CCTGTGGCCTTGATGGAGAG-3'
	R: 5'-TCTCTCCTTTGTGTCCTGCC-3'
Human RNASE2 primer	F: 5'-GAGGGCTCACTCCATGTCAA-3'
	R: 5'-ATTGGTGCATTGCTGGGAGG-3'
Human SLC10A2 primer	F: 5'-TGAAACGGGGATGCAGAACA-3'
	R: 5'-TGAGCGGGAAGGTGAATACG-3'
Human APOLD1 primer	F: 5'-CTCGTTCACGGATGTTCCGC-3'
	R: 5'-CCATTCCCTTTCCAAGGCAGG-3'
Human β-actin primer	F: 5'-CCAACCGCGAGAAGATGACC-3'
	R: 5'-GAGTCCATCACGATGCCAGT-3'
